# The effectiveness of Reslizumab in severe asthma treatment: a real-world experience

**DOI:** 10.1186/s12931-019-1251-3

**Published:** 2019-12-20

**Authors:** H. Ibrahim, R. O’Sullivan, D. Casey, J. Murphy, J. MacSharry, B. J. Plant, D. M. Murphy

**Affiliations:** 1 The Department of Respiratory Medicine, Cork University Hospital, Cork, Ireland; 2 The Schools of Medicine and Microbiology, University College Cork, Cork, Ireland; 3 The HRB funded Clinical Research Facility, University College Cork, Cork, Ireland

**Keywords:** Anti-IL5, Asthma, Eosinophils

## Abstract

**Background:**

Increased numbers of blood and sputum eosinophils are associated with higher exacerbation frequency and increased asthma severity. In clinical trials, targeting Interleukin-5 has been shown to be a useful therapeutic strategy for patients with severe eosinophilic asthma.

**Methods:**

Twenty-six patients have been commenced on Reslizumab in our institution since early 2017. Safety and clinical efficacy parameters were recorded at regular intervals.

**Results:**

Mean ACQ-6 score at the start of treatment was 3.5. The average number of exacerbations in the year preceding treatment was 8.3 per person. 30% of patients had been admitted to hospital at least once over the 12 months preceding therapy. 54% of our patients were on long term oral steroid.

Our data showed sustained improvement of Asthma control (Mean improvement in ACQ-6 was 1.7 at 1 year, and 2.0 at 2 years, *P* = 0.0001). Of the patients who were on long term systemic steroids, 35.7% discontinued steroids completely, with a mean reduction of prednisolone dose of 5.2 mg at 1 year. There was a 79% reduction in the annual exacerbation frequency at 1 year, and 88% at 2 years (*P* = < 0.0001). Modest, albeit statistically significant increases in creatine kinase which seemed to plateau by 1 year were noted.

**Conclusions:**

Overall, Reslizumab was well tolerated with discontinuation of treatment due to side effects recorded in only one patient. Our data confirm the utility of anti-IL5 therapy in a carefully selected phenotype of severe asthma with evidence of eosinophilic airway inflammation.

## Background

A subset of patients with asthma remain uncontrolled with conventional therapy. Approximately 5–10% of asthmatics have severe asthma [1], often requiring high dose inhaled glucocorticoids, and/or systemic glucocorticoids. Almost half of patients with severe asthma require regular or frequent courses of systemic steroids to control their disease, with some never achieving optimal control of their condition [2]. Severe asthma with eosinophilia is a phenotype of severe asthma characterized by increased numbers of circulating and airway eosinophils. Increased numbers of blood and sputum eosinophils are associated with higher exacerbation frequency [3] and increased asthma severity [4].

Interleukin-5 [IL-5] is a major regulator of eosinophil survival and activity in tissues. It has been shown to mediate late stage maturation of eosinophils from eosinophil lineage-committed progenitors through IL-5R [5]. IL-5 also affects eosinophils activation and effector function, and prevents apoptosis of mature eosinophils with prolongation of eosinophil survival [6].

In clinical trials, targeting IL-5 has been shown to be a useful therapeutic strategy for patients with severe asthma. Currently available anti IL-5 monoclonal antibodies are benralizumab, reslizumab and mepolizumab. Benralizumab, a monoclonal antibody directed against the alpha subunit of the IL-5 receptor, has shown significant and clinically relevant reduction in the dose of oral glucocorticoids and a significant reduction in exacerbation rate [7]. Mepolizumab treatment in severe asthma has shown significant reduction in exacerbation frequency, with significant improvements in lung function and asthma symptoms [8].

Reslizumab is a humanized monoclonal antibody directed against Interleukin-5. It binds specifically to IL-5 thereby blocking its biological effects including recruitment and activation of human eosinophils. Clinical trials have shown that reslizumab treatment in patients with severe asthma and evidence of peripheral eosinophilia was well tolerated and provided improvement in terms of lung function, clinical asthma exacerbation, and quality of life [9–11]. Here we report our experience with Reslizumab treatment in clinical practice.

## Methods

Twenty-six patients have been commenced on reslizumab in our institution since early 2017.

This was an early access programme for patients. At a minimum inclusion criteria were; inadequately controlled asthma (ACQ-6 score > 1.5), elevated peripheral blood eosinophils count (> 0.4 cells X 10^9^/L), on high dose inhaled steroids and a second controller, and had at least 4 exacerbations (that required systemic steroids or an increase in maintenance steroid dose), 1 hospitalisation or a requirement for maintenance oral steroid for > 6 months over the year preceding treatment. As this was an early access programme the majority of our cohort had more severe asthma than these criteria.

Reslizumab was administered as an intravenous [IV] infusion (3 mg/kg) every 4 weeks and in accordance with the product license. Reslizumab was provided by Teva Pharmaceuticals as part of a named patient / early access programme. Asthma control (ACQ), glucocorticoid dose, exacerbation history, and FEV1 were recorded prior to commencing treatment and at regular intervals to assess clinical effectiveness as is standard practice in our institution.

## Results

### Baseline demographic and clinical characteristics

The mean age at the start of treatment was 52 years (*SD* ± 13.5). 62% of patients were female. Mean ACQ-6 score at the start of treatment was 3.5 (*SD* ± 1.1), average percent of forced expiratory volume in 1 s (FEV_1_) predicted value was 62% (*SD* ± 20%), and average absolute peripheral blood eosinophil count was 0.79 (*SD* ± 0.52) cells X 10^9^/L.

54% of our patients were on long term maintenance oral steroid. The mean steroid dose amongst patients on long term glucocorticoids was 9.3 (*SD* ± 4.3) mg. The average number of exacerbations in the year preceding treatment (that required systemic steroids or an increase in maintenance steroid dose) was 8.3 per person. 30% of patients had been admitted to hospital at least once over the 12 months preceding therapy. (See Table 1).

**Table 1:** Baseline demographic and clinical characteristics

Patients and disease variables	Baseline (*N* = 26)
The mean age	52 years (SD ± 13.5).
Mean ACQ-6 score ± SD	3.5 (SD ± 1.1)
Systemic glucocorticoid.	54% were on maintenance oral steroid^a^
Mean steroid dose amongst patients on long term glucocorticoid^b^	9.3 (SD ± 4.3) mg^b^
Mean percent of FEV_1_ predicted value (before bronchodilation) + − SD	62% (SD ± 19.9)
Average number of exacerbations^c^ ± SD	8.3 (SD ± 4.7)
Mean peripheral blood Eosinophils count^d^ ± SD	0.78 cells X 10^9^/L (SD ± 0.51)

*ACQ-6* Asthma Control Questionnaire, *FEV1* Forced Expiratory Volume in 1 second, ^a^*N*=14, ^b^Prednisolone dose in mg, ^c^Average number of exacerbations per year that required rescue systemic steroids course or increase in the maintenance steroid dose, ^d^cells X 10^9^/L

### Asthma control and lung function

There were significant improvements in asthma symptoms and control assessed using ACQ-6 score at baseline and after 3 months on treatment, with mean ACQ-6 score improved from 3.5 at base line to 1.8 at 3 months (*P* < 0.0001).

**Fig. 1:**
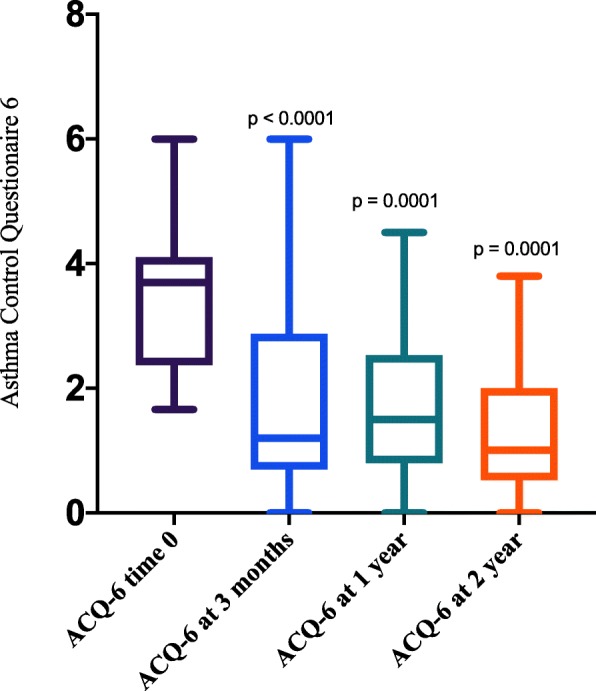
Whisker Plot comparing ACQ-6 at baseline, 12 weeks, 1 and 2 years post treatment

Long term data showed sustained improvement in Asthma control in the subgroup of patients completing 1 year [22 patients] & 2 years [11 patients] of treatment (Mean improvement in ACQ-6 was 1.7 at 1 year, and 2.0 at 2 years, *P* = 0.0001). (See Fig. 1).

Improvement in lung function wasn’t statistically significant at 3 months, but at 1 year of treatment the mean improvement in FEV-1% of predicted value was 11.9% (*P* = 0.018), and mean improvement was 12.1% at 2 years (*P* = 0.002) (See Table 2).

**Table 2:** Summary of variables at baseline, 12 weeks, 1 year and 2 years of treatment

Variable*s*	Baseline (N=26)	12 weeks (N=24)	1 year (N=22)	2 year2 (N=11)
Mean ACQ-6 score ± SD	3.50 ± 1.1	1.8 ± 1.7	1.7 ± 1.4	1.3 ± 1.1
Mean improvement in ACQ-6	*—*	1.7	1.7	2.0
P value	*—*	<0.0001	0.0001	0.0001
Mean glucocorticoid dose^a^ ± SD	9.29 ± 4.32	4.79 ± 4.07	4.77 ± 4.53	4.62 ± 3.59
Median reduction in the final oral glucocorticoid-% of baseline value	*—*	50%	50%	50%
Mean reduction of prednisolone dose ± SD	*—*	4.50 ± 3.85	5.23 ± 4.80	4.12 ± 4.06
P Value	*—*	0.0008	0.0048	0.0239
Mean percent of FEV_1_ predicted value *(before bronchodilation)* +- SD	62.08% ± 19.85	64.51% ± 25.30	70.61% ± 15.40	74.91 ± 22.63
Mean improvement in FEV1 percent of predicted value	*—*	6.28	11.95	12.14
P value	*—*	0.36	0.018	0.0021
Average number of exacerbations^b^ *± SD*	8.32 ± 4.68	*—*	1.74 ± 2.13	0.91 ± 0.70
Mean reduction in annual Exacerbations^b^ ± SD	*—*	*—*	7.26 ± 4.74	6.64 ± 3.29
P Value	*—*	*—*	<0.0001	<0.0001
Mean peripheral blood Eosinophils count^c^ ± SD	0.78 ± 0.51	0.05 ± 0.03	0.05 ± 0.04	0.04 ± 0.03
Mean reduction of eosinophils counts	*—*	0.73 ± 0.52	0.74 ± 0.57	0.72 ± 0.63
P Value	*—*	<0.0001	<0.0001	0.0057

*ACQ-6* Asthma Control Questionnaire, *FEV1* Forced Expiratory Volume in 1 second

^a^Prednisolone dose in mg

^b^Average number of exacerbations per year that required rescue systemic steroids course or increase in the maintenance steroid dose

^c^cells X 10^9^/L

### Asthma exacerbations and steroid dose reduction

Of the 14 patients who were on long term systemic steroids, 35.7% discontinued steroids completely, with a mean reduction of prednisolone dose of 5.2 mg among patients who completed 1 year of treatment. In the subgroup of patients who completed 2 years of treatment the mean reduction was 4.6 mg (50% of baseline value), No further improvement was noted at 2 years of treatment compared to 1 year (See Table 2).

**Fig. 2:**
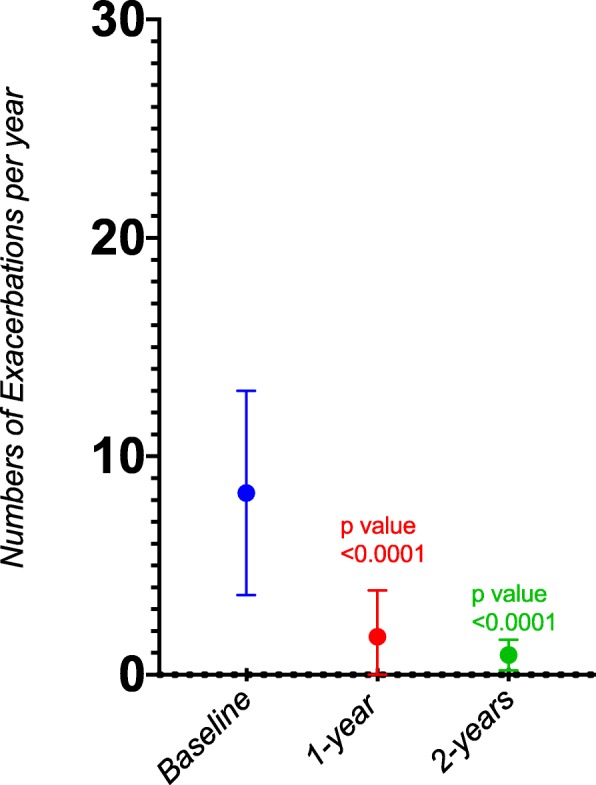
Annual Exacerbations at baseline, 1 year & 2 years post treatment

The average number of exacerbations in the year preceding treatment (that required a course of rescue systemic steroids or increase in the maintenance steroid dose) was 8.3 per person. There was a 79% reduction in the annual exacerbation frequency in the patients who completed 1 year of treatment, with 47% having no exacerbations. (*P* = < 0.0001, Mean –reduction 7.3, 95% confidence interval 9.6 to 5). Furthermore, there was 88% reduction in the annual exacerbation frequency in the patients who completed 2 years of treatment (See Fig. 2).

Predictably, treatment with a humanized monoclonal antibody directed against Interleukin-5 resulted in a significant reduction in peripheral blood eosinophil count. (*P* < 0.0001) (See Table 2).

### Safety and side-effect

Resluzimab has been generally well tolerated amongst our patients. The most common side-effects reported have been fatigue and we have observed elevations of creatinine kinase level (Mean creatine kinase level increased from 94.1 U/L pretreatment level to 184.7 U/L after 3 months of therapy (p = 0.025), and 160.5 U/L at 1 year (p = 0.031). The normal range for creatine kinase in our institution is 40–180 U/L.

Only one patient has discontinued treatment due to an adverse event [AE] - an allergic skin rash which disappeared after cessation of reslizumab.

Treatment discontinued in 5 other patients. One patient, although treatment resulted in a significant improvement in her asthma control, reslizumab was discontinued as she was actively planning to attempt to conceive. In 4 patients treatment was withdrawn due to lack of therapeutic benefit.

## Discussion

Our real-world data confirm the positive findings of clinical trials [9–11]. Improvements in asthma control assessed using a validated asthma control questionnaire was statistically significant (Mean improvement in ACQ-6 was 1.7 at 3 months compared to a mean improvement of 0.8 at 16 weeks in clinical trials) [9]. Furthermore, reslizumab had a steroid sparing effect, with significant reductions in maintenance steroid doses. The response was noted within 12 weeks of treatment and sustained in the group of patients who have completed 2 years of treatment. (The median reduction in oral glucocorticoid dose was 50% at 2 year of treatment). Benralizumab showed a median reduction in oral steroid dose of 75% at 28 weeks of therapy [7].

Our 2 years data showed a significant reduction in asthma exacerbations (88% reduction in patients who have completed 2 years of treatment), noting reslizumab Phase 3 clinical trials in poorly controlled asthma were not designed to assess asthma exacerbations as an end point given the short duration of the clinical trials [9, 10]. A 52 weeks open label extension study from phase 3 clinical trial has shown a 50% reduction in clinical asthma exacerbations compared to placebo [12].

While small improvements in lung function were noted in patients on resluzimab after 3 months these were not significant, but both 1 year and 2 year data showed significant improvement in lung function (mean improvement in FEV-1% of predicted value was 11.9% at 1 year and 12.1% at 2 years). This suggests that the largest improvements in FEV-1 are within the first 12 months of treatment although maintained thereafter.

Overall, Reslizumab was well tolerated with discontinuation of treatment due to side effects recorded in only one patient. Modest, albeit statistically significant increases in creatine kinase which seemed to plateau by 1 year were noted. The exact aetiology of this increase is unclear. The subgroup of 4 patients who displayed no clinical response to therapy had more frequent exacerbations (10.7 per year vs 8.3), worse lung function (FEV1 49% vs 62%), and baseline asthma control (ACQ 4.3 vs 3.5) compared to the overall study group. Baseline eosinophil count was similar to the studied group, and interestingly none of these patients were on long term steroids. The treatment resulted in significant depletion of eosinophils in these patients, but this wasn’t reflected by a clinical response suggesting that poor asthma control in this subgroup of patients wasn’t entirely driven by eosinophilic airway inflammation.

Our data confirm the utility of anti-IL5 therapy in a carefully selected phenotype of severe asthma with evidence of eosinophilic airway inflammation.

## Data Availability

The datasets generated during and/or analyzed during the current study are available from the corresponding author on reasonable request.

## Abbreviations

ACQ: Asthma Control Questionnaire
ACQ-6: Asthma Control Questionnaire 6
AE: Adverse event
CI: Confidence interval
FEV1: Forced Expiratory Volume in one second
IV: Intravenous
SD: Standard deviation

## References

[1] Chung KF, Wenzel SE, Brozek JL, Bush A, Castro M, Sterk PJ (2014). International ERS / ATS guidelines on definition , evaluation and treatment of severe asthma. Eur Respir J.

[2] Shaw DE, Sousa AR, Fowler SJ, Fleming LJ, Roberts G, Corfield J (2015). Clinical and inflammatory characteristics of the European U-BIOPRED adult severe asthma cohort. Eur Respir J.

[3] Zeiger RS, Schatz M, Li Q, Chen W, Khatry DB, Gossage D (2014). High blood eosinophil count is a risk factor for future asthma exacerbations in adult persistent asthma. J Allergy Clin Immunol Pract.

[4] Price DB, Rigazio A, Campbell JD, Bleecker ER, Corrigan CJ, Thomas M (2015). Blood eosinophil count and prospective annual asthma disease burden : a UK cohort study. Lancet Respir.

[5] Sitkauskiene B, Johansson A, Sergejeva S, Lundin S, Sjo M, Lo J (2004). Regulation of bone marrow and airway CD34+ Eosinophils by Interleukin-5. Am J Respir Cell Mol Biol.

[6] Rosenberg HF, Phipps S, Foster PS (2007). Eosinophil trafficking in allergy and asthma. J Allergy Clin Immunol.

[7] Nair P, Wenzel S, Rabe KF (2017). Oral glucocorticoid–sparing effect of Benralizumab in severe asthma. N Engl J Med.

[8] Ortega HG, Liu MC, Pavord ID, Brusselle GG, Fitzgerald JM, Chetta A (2014). Mepolizumab treatment in patients with severe Eosinophilic asthma. N Engl J Med.

[9] Bjermer L, Lemiere C, Maspero J, Weiss S, Zangrilli J, Germinaro M (2016). Reslizumab for inadequately controlled asthma with elevated blood eosinophil levels. Chest.

[10] Corren J, Weinstein S, Janka L, Zangrilli J, Garin M (2016). Phase 3 study of Reslizumab in patients with poorly controlled asthma, effects across a broad range of eosinophil counts. Chest.

[11] Castro M, Zangrilli J, Wechsler ME, Bateman ED, Brusselle GG, Bardin P (2015). Reslizumab for inadequately controlled asthma with elevated blood eosinophil counts: results from two multicentre, parallel, double-blind, randomised, placebo-controlled, phase 3 trials. Lancet Respir Med.

[12] Murphy K, Jacobs J, Bjermer L, Fahrenholz JM, Shalit Y (2017). Long-term Safety and Efficacy of Reslizumab in Patients with Eosinophilic Asthma. J Allergy Clin Immunol Pract.

